# Mineral exploration and environmental impact assessment in the Jabal Hamadat Area, Central Eastern Desert, Egypt, using remote sensing and airborne radiometric data

**DOI:** 10.1038/s41598-024-71387-2

**Published:** 2024-09-23

**Authors:** Mahmoud Abd El-Rahman Hegab

**Affiliations:** National Authority for Remote Sensing and Space Sciences (NARSS), Cairo, Egypt

**Keywords:** Hydrothermal alteration, Environmental pollutions, Jabal Hamadat, Radiological hazard, Environmental impact, Geophysics

## Abstract

Mineral exploration is essential for economic growth, but it must be conducted with careful consideration of health and environmental impacts. This study focuses on mapping potential mineralization areas and evaluating environmental consequences in the Jabal Hamadat area. By integrating ASTER, Landsat-9 and airborne radiometric data, the study includes: (1) identification of alteration mineral indicators including iron oxides (gossanized zone), chlorite (propylitic zone), kaolinite, sericite, montmorillonite/illite (argillic zone), and alunite (advanced argillic zone) via the Band Ratio (BR) technique; (2) preparation of a lineament density map through an automated lineament extraction technique; and (3) identification of areas with elevated F-parameter values exceeding 10. Ten areas with high mineralization potential are delineated for further exploration. Additionally, the study assesses environmental radiation exposure, finding that certain areas exceed the safe radiation limit of 1.0 mSv/year. Peak radiation levels range from 0.75 to 1.25 mSv/year, with mining sites showing the highest readings at 1.25 mSv/year and 0.64 mSv/year. These findings highlight elevated radiological hazards, emphasizing the need for comprehensive monitoring and effective mitigation strategies to protect human health and minimize environmental impact. The methodology's success in this area indicates its potential applicability to other mining areas, contributing to enhanced safety and environmental protection.

## Introduction

The Eastern Desert of Egypt yields a diverse array of mineral deposits across its expansive terrain. Geologically, it forms part of the esteemed Arabian-Nubian Shield, renowned for its extensive metamorphic and igneous rock formations dating back to the Precambrian era^1,2^. Within this shield lie ancient crystalline structures like granites, gneisses, and schists, forged through dynamic tectonic processes like mountain building and volcanic eruptions^3^. During the Neoproterozoic era, the Eastern Desert experienced profound volcanic and plutonic activities, laying the foundation for the emergence of numerous mineral reserves, including coveted resources like gold, copper, and various base metals^4,5^. This intricate geological history underscores the region's significance as a prime destination for mineral exploration and extraction endeavors.

Remote sensing satellite imagery proves invaluable in detecting and mapping hydrothermal alteration zones, greatly aiding mineral exploration^6–10^. Utilizing multispectral satellite images like ASTER and Landsat-9 is common due to their ability to capture radiation across multiple spectral bands, covering a wide range of wavelengths essential for mineral detection^11–14^. This capability facilitates the characterization of mineralogical compositions by analyzing the absorption, reflection, and emission properties of various minerals across diverse wavelengths^15–17^. Alteration minerals exhibit distinct absorption features in specific wavelength regions, notably in the VNIR range (0.3 to 1.0 μm) and the SWIR range (2.0 to 2.5 μm), serving as diagnostic indicators for their identification and characterization^8,18,19^. These alteration zones can manifest in various geological settings, including volcano-plutonic rocks and hydrothermal mineral deposits, which offer significant potential for mineral exploration^20–23^. The distinctive mineral assemblages shaped by hydrothermal alteration processes in these areas serve as critical guides for mineral exploration and the identification of potential mining sites^16–18,24,25^.

The utilization of airborne radiometric data has emerged as a powerful technique for delineating hydrothermal alteration zones, involving the assessment of concentrations of K, eU, and eTh within three broad spectral windows^26^. Understanding the radioactivity of soils in anomalous radioactive areas is critical for comprehending changes in the natural radiation background^27,28^. By studying the concentrations of radioelements, valuable insights into the formation and evolution of rocks, as well as the geological processes shaping the Earth's surface, can be gained. This knowledge of radioelement distribution is pivotal for mineral exploration, aiding in the identification of areas with high mineral potential^29–31^. Furthermore, understanding the distribution of radioelements in rocks is indispensable for environmental studies, providing information about potential sources of radiation and their environmental impact^32–34^.

The Central Eastern Desert of Egypt has witnessed significant volcanic and plutonic activities, which is important for hosting potential mineralized zones and mining activities^9,25,29,34–36^. These activities have laid the foundation for the mineralization of gold, copper, and various base metals^13,19,37–39^. However, they also pose serious environmental threats, affecting the health and well-being of the human population living near or adjacent to mining areas. Therefore, the Jabal Hamadat area was selected for the present study to identify potential mineralized zones for exploration and to assess the corresponding radiological hazards and environmental impacts using an innovative approach by integrating remote sensing and airborne radiometric data.

## Study area and geology

**Fig. 1:**
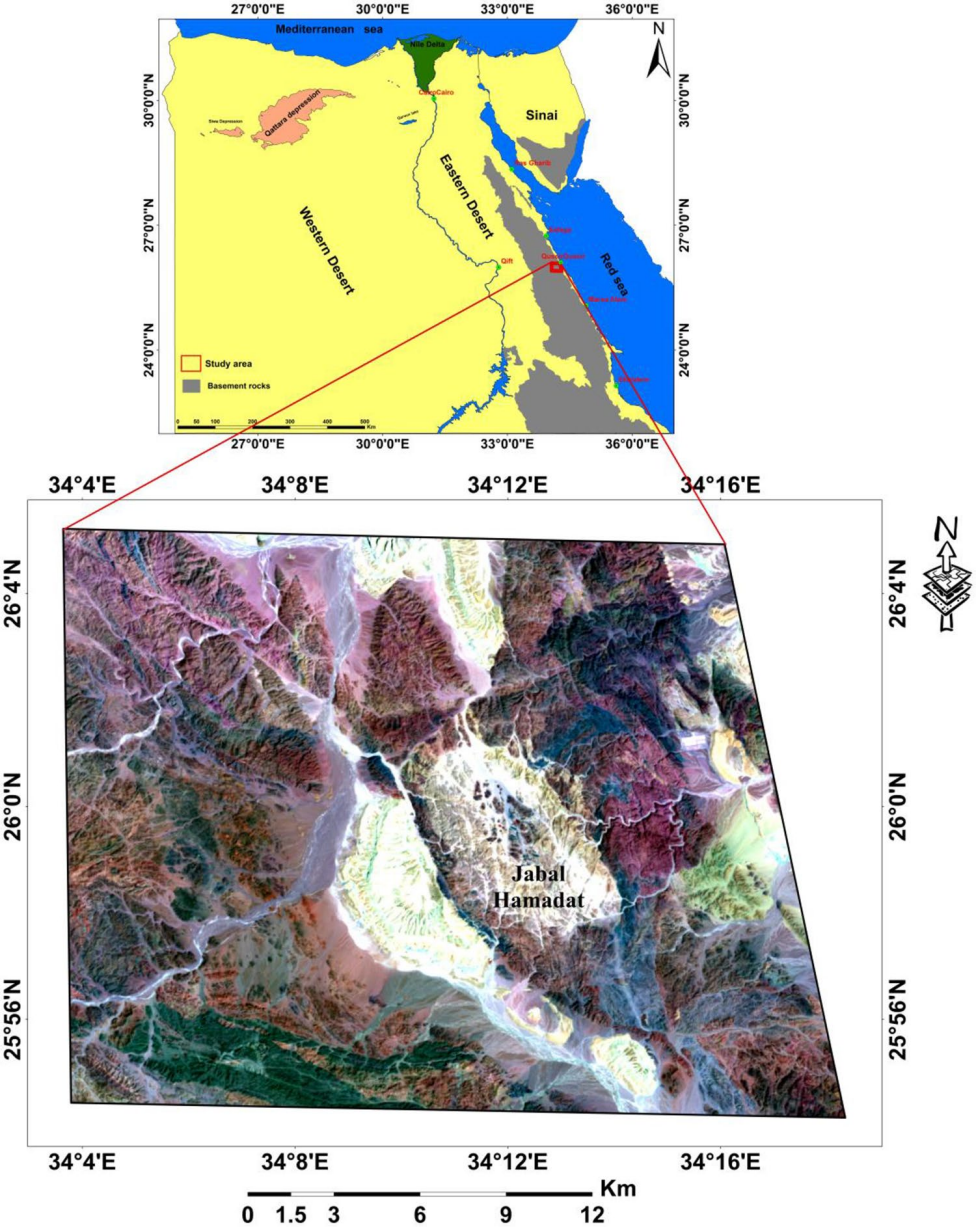
Location map of Jabal Hamadat area, Central Egyptian Eastern Desert. (By ArcGIS v.10.5. https://www.esri.com/en-us/arcgis/products/arcgis-desktop/overview/).

The Jabal Hamadat area is located in the Central Eastern Desert of Egypt, positioned on the western side of the red sea margin. It lies approximately between latitudes 25°54' and 26°05' and longitudes 34°03' and 34°18' (Fig. 1).

**Fig. 2:**
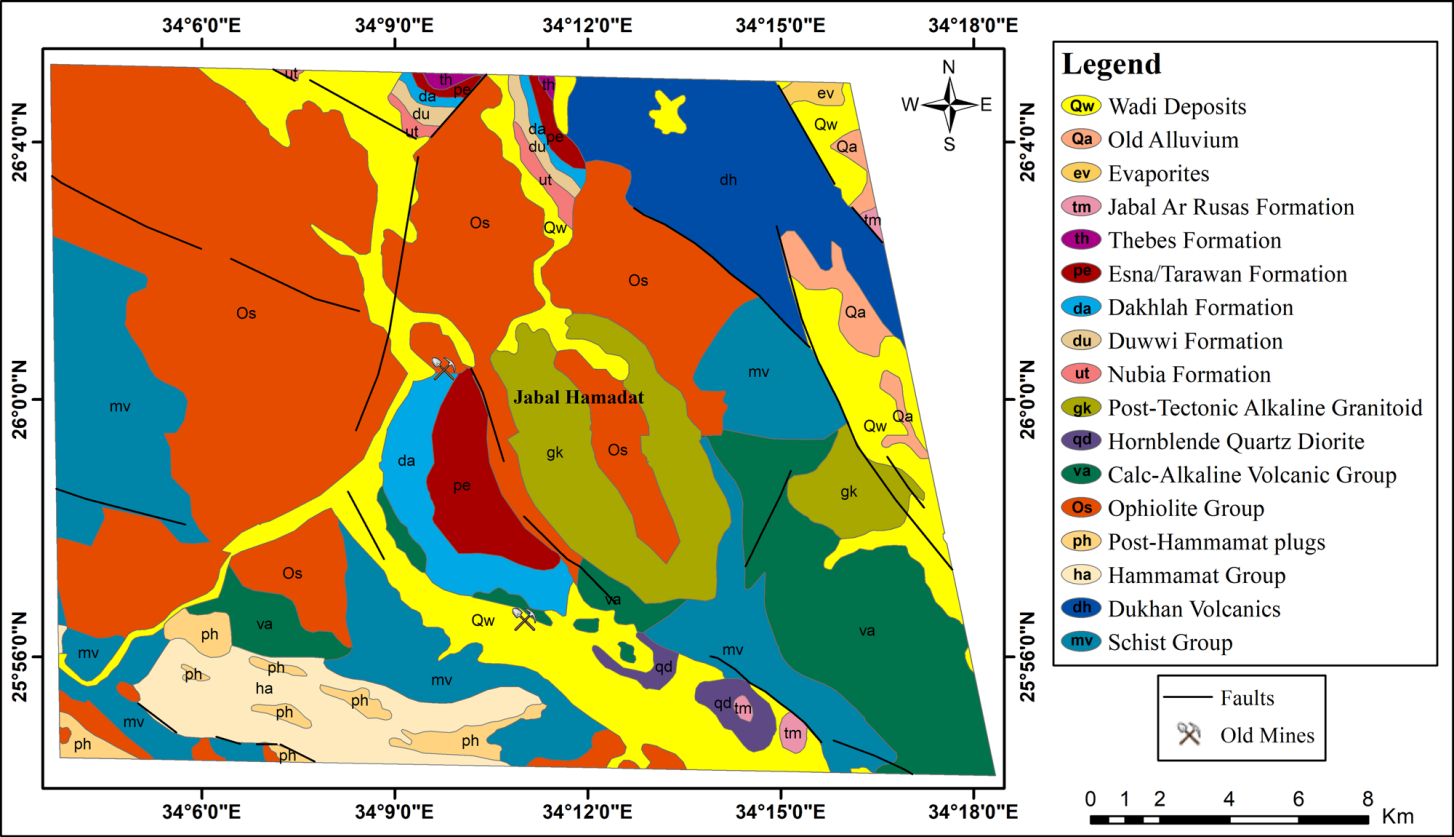
Geological map of Jabal Hamadat area, Central Egyptian Eastern Desert (modified after EGSMA^40^). (By ArcGIS v.10.5. https://www.esri.com/en-us/arcgis/products/arcgis-desktop/overview/).

The geological formations in the study area encompass diverse lithological units and depositional settings. The rock units exposed in the Jabal Hamadat area are presented in chronological order (Fig. 2), with the youngest formations listed as follows: (1) Recent wadi deposits: consisting of beach and wadi gravel deposits, silt and sandy bands, and remnants of coral reefs. (2) Old alluvium: featuring limestone and chert pebbles, cobbles with calcareous cement, and thin maristone beds. (3) Evaporites: comprising steep-sloped gypsum and anhydrite beds, along with green-grey shale and mari intercalations. (4) Jabal Ar Rusas formation: characterized by conglomerate, sandstone, and shale, with occasional mari and limestone bands. (5) Thebes formation: exhibiting pale white porcellanic chalky limestone, brown/black flint bands and nodules, and fossiliferous limestone (oyster beds). (6) ESNA formation: consisting of green glauconite-bearing clays and shales with limestone interbeds. (7) Tarawan formation: featuring chalk with beds of black chert. (8) Dakhlah formation: comprising green and blue-grey laminated shales, minor limestone, and calcareous mudstone rich in Pecten farafrensis, along with veins of fibrous gypsum and calcite. (9) Duwwi formation: including clays, calcareous mudstone, phosphates, and siliceous mudstone bearing Ostrea villei. (10) Nubia formation: composed of qusayr clastics and tarif sandstone, with characteristics such as thinly bedded variegated clays and shales, siltstones, cross-bedded sandstones, and pebble beds. (11) Post-tectonic alkaline granitoid: featuring red-pink coarse-grained biotite ± muscovite alkali granite and pink-grey coarse-grained biotite ± hornblende granite with abundant amphibolite, metavolcanic xenoliths, and porphyritic felsite sheets. (12) Hornblende quartz diorite: consisting of coarse and medium-grained hornblende gabbro and hornblende diorite. (13) Calc-alkaline volcanic group: comprising reworked pyroclastic and volcaniclastic deposits, aphyric intermediate to basic lavas, and acidic to intermediate aphyric lavas, among other characteristics. (14) Ophiolite group: featuring talc, ankerite, graphite, tremolite serpentinite metavolcanic schist, mesh-textured serpentinite asbestos, and magnesite. (15) Post-hammamat plugs: including dark-grey to light-khaki brown medium-grained quartz, quartz feldspar plugs, and dyke-like plugs. (16) Hammamat group: characterized by purple-red poorly sorted molassoid clastics, polymictic boulders and cobble conglomerate, pebbly and massive greywackes, and alternating red and green siltstone and mudstone. (17) Dukhan volcanic: comprising quartz-feldspar porphyrite subvolcanics and plagioclase-phyric andesite. (18) Schist group: featuring biotite sericite schist and quartz schist with minor ironstone and thin tectonic sheets of talc, ankerite, and graphite lenses.

## Data and methodology

### Data used

The study utilized a comprehensive dataset, including Landsat-9 and ASTER images, which included 11 and 14 spectral bands, respectively (Tables 1 and 2). Additionally, airborne radiometric data, involving measurements of radiation collected from the Earth's surface using specialized sensors mounted on airborne platforms, was incorporated into the analysis.

**Table 1:** Landsat-9 sensor specifications contribution to this research (Landsat-9 user’s handbook):

Band number	Wavelength range (μm)	Resolution (meters)	Band description	Contribution
1	0.43–0.45	30	Coastal/Aerosol	✗
2	0.45–0.51	30	Blue	✓
3	0.63–0.69	30	Green	✓
4	0.77–0.89	30	Red	✓
5	1.55–1.75	30	Near-Infrared (NIR)	✓
6	2.11–2.29	30	Short-Wave Infrared (SWIR) 1	✓
7	2.29–2.53	30	Short-Wave Infrared (SWIR) 2	✓
8	0.50–0.68	15	Panchromatic	✗
9	1.36–1.38	30	Cirrus	✗
10	10.60–11.19	100	Thermal Infrared (TIRS) 1	✗
11	11.50–12.51	100	Thermal Infrared (TIRS) 2	✗
12	2.50–2.70	30	Short-Wave Infrared (SWIR) 3 (Quality Band)	✗

**Table 2:** ASTER sensor specifications contribution to this research (ASTER user’s handbook).

System	Band numbers	Band width (μm)	Spatial resolution	Contribution
VNIR (Visible and Near-Infrared)	1 (0.52–0.60 μm), 2 (0.63–0.69 μm), 3 (0.78–0.86 μm)	0.08–0.18 μm	15 m	✓
SWIR (Shortwave Infrared)	4 (1.60–1.70 μm), 5 (2.145–2.185 μm), 6 (2.185–2.225 μm), 7 (2.235–2.285 μm), 8 (2.295–2.365 μm), 9 (2.360–2.430 μm)	0.04–0.08 μm	30 m	✓
TIR (Thermal Infrared)	10 (8.125–8.475 μm), 11 (8.475–8.825 μm), 12 (8.925–9.275 μm), 13 (10.25–10.95 μm), 14 (10.95–11.65 μm)	0.35–1.4 μm	90 m	✗

Cloud-free multi-sensor Landsat-9 imagery was obtained from the U.S. Geological Survey Earth Resources Observation and Science Center (EROS) (https://earthexplorer.usgs.gov/), while ASTER imagery acquired on 7–2-2004 was sourced from NASA Earth Observing System (EOS) (https://www.earthdata.nasa.gov/). Table 1 provides the technical specifications of the Landsat-9 sensor, including corresponding bands suitable for this research. Similarly, Table 2 outlines the technical specifications of the ASTER sensor, along with corresponding bands suitable for this study. Both sensors complement each other, covering specific portions of the spectrum, which enables comprehensive geological and mineralogical research.

The Aero-Service Report (1984) provides detailed specifications for airborne radiometric data collection. The survey uses a gamma-ray spectrometer with a 256-channel detector array, including primary detectors for secondary radiation and specialized detectors for airborne radon. The flight traverses are conducted at angles of 45/225 degrees and 135/315 degrees, with a terrain clearance of 120 m. Flights maintain an altitude of 1.5 km for traverses and extend to 10 km for tie lines. The flight intervals are set at 0.1%, 0.5%, and 5.0%, and the contour interval is one-tenth of 1 K. These parameters ensure precise and comprehensive data collection, as outlined in the report^41^.

### Methodology

#### Remote sensing data processing

**Fig. 3:**
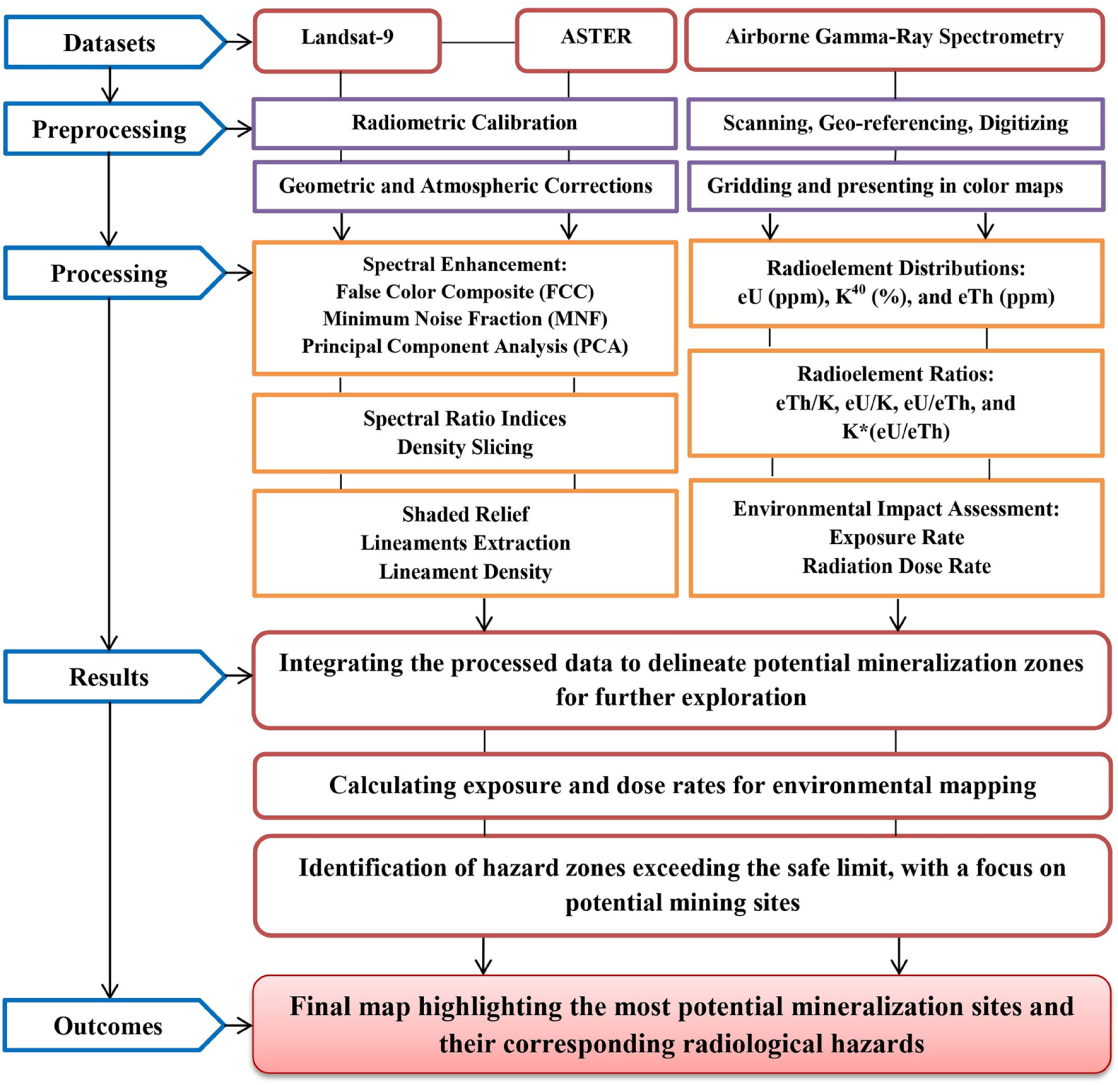
The methodology flowchart.

The logical framework of the methodology adopted in this research is shown in Fig. 3. In the analysis of remote sensing satellite data, several preprocessing and analysis steps have been implemented. ENVI 5.3 and Arc GIS 10.5 were used to manipulate, preprocess, and process this data. Initially, FLAASH atmospheric correction was applied to alleviate the influence of atmospheric interference on the image quality. Following this, the VNIR and SWIR bands were stacked, and wavelength definitions were established for each band. Subsequently, spectral enhancement techniques, including FCC, MNF, and PCA, were employed on Landsat-9 data to discern between various rock types effectively. These methods provide valuable insights into the main lithological units^42–44^.

Additionally, we utilized the BR technique on ASTER data to accurately quantify the abundance of alteration minerals across the study area. ASTER has extensive capabilities for identifying altered minerals, such as kaolinite, montmorillonite, illite, sericite, alunite, chlorite, epidote, and calcite^45^. The BR technique is highly demonstrated in mapping the mineral/rock alterations^13,19,37–39^. This technique involves dividing the digital number (DN) values of one band by those of another, enhancing specific features that may not be easily discernible in raw bands alone^37^. By minimizing differences resulting from albedo and topographic slope effects, the BR method effectively enhances compositional variation, thereby accentuating the spectral characteristics of rocks and minerals^37,38,46^. Our findings highlight the effectiveness of the BR method in extracting information on hydrothermal alteration minerals, as demonstrated in Table 3. Furthermore, we applied the density slicing technique to ratio images, which further refined our results by highlighting various alteration minerals within the study area. This method segments grayscale values from 0 to 255 in single-band images into intervals or slices, each assigned a distinct color, thereby refining the results and filtering out extraneous information.

**Table 3:** The mineral indices adopted through the BR technique.

Mineral	Band Ratio	Reference
Iron oxides	Band 2/Band 1	^47^
Montmorillonite/Illite	(Band 5 + Band 7)/Band 6	^48^
Kaolinite	(Band 4/Band 5) * (Band 8/Band 6)	^49^
Chlorite	(Band 7 + Band 9)/Band 8	^48^
Alunite	(Band7/Band5) * (Band 7/Band 8)	^49^
Sericite	Band 7/Band 6	^48^

The automated lineament extraction technique was then utilized to identify lineaments, map linear features, and enhance the understanding of the structural framework within the study area. This automated process proves valuable for delineating the distribution of hydrothermal alteration features, thus providing critical insights into potential mineralized zones^24,50^.

#### Airborne radiometric data analysis

The airborne radiometric data analysis involves gamma radiation detected by an aerial gamma-ray spectrometer (AGRS), originating from three primary sources: 1) K-40, constituting 0.0118% of total potassium; 2) U-238 decay series daughter products; and 3) Th-232 decay series daughter products. Following geo-referencing for spatial analysis, the Jabal Hamadat area of interest is delineated, digitized, and gridded. Subsequently, maps depicting the distribution of radioelements (K, eU, eTh), radioelement ratios (eTh/K, eU/K, and eU/eTh), and the F-parameter map (K*(eU/eTh)) are generated. This process aims to identify radiation signatures associated with different elements or minerals, facilitating the recognition of potential mineralization and contributing to a comprehensive understanding of the study area.

Regarding the environmental monitoring, estimating natural gamma radiation (exposure rate) is valuable as it often has the most significant impact on environmental contamination. Subsequently, the equivalent radiation dose rate was determined based on the radiation exposure rate, providing insights into potential hazards to human health and various effects on biological tissues. The strength of radiation at a specific location is termed "Exposure" (E), measured by its capacity to induce ionization in that area. The ground level exposure rate can be calculated using the apparent concentrations of K (%), eU (ppm), and eTh (ppm), as outlined by the IAEA (2003), with the formula^26^:

1 $${\text{E }}\left( {\mu {\text{R}}/{\text{h}}} \right) \, = { 1}.{5}0{\text{5 K }}\left( \% \right) \, + \, 0.{\text{653 eU }}\left( {{\text{ppm}}} \right) \, + \, 0.{\text{287 eTh }}\left( {{\text{ppm}}} \right).$$

It's essential to recognize that the exposure rate calculated using this equation accounts solely for gamma-ray exposure from radioactive sources in the ground, excluding the cosmic-ray component or any cesium fallout on the ground. The unit of "absorbed energy" or "Dose" (D) represents the energy imparted by ionizing radiation to one gram of any material at the specific point of interest. The rem (roentgen equivalent man) serves as the unit of absorbed dose, with one rem indicating the dose from any radiation that produces biological effects in a human. The conversion from exposure rate to dose rate is determined by:

2 $${\text{D }}\left( {{\text{millirem }}/{\text{ year}}} \right) \, = {8}.{33}*{\text{E }}\left( {\mu {\text{R}}/{\text{h}}} \right).$$

In contemporary practice, measurements related to radiation protection are commonly conveyed in SI units. The Sievert (Sv) serves as the standard SI unit for equivalent doses, analogous to the rem^51^. This relationship is expressed as follows:

3 $${\text{1 Sv }} = { 1}00{\text{ rem}},$$

4 $${\text{Then}};{\text{ D }}\left( {{\text{mSv }}/{\text{ year}}} \right) \, = \, 0.0{833 }*{\text{ E }}\left( {\mu {\text{R}}/{\text{h}}} \right).$$

The IAEA (2005) guidelines categorize radiation dose equivalent (RDE) as follows^52^: If RDE is expected to stay below 1 mSv annually, minimal intervention is needed to assess and manage worker doses. For RDE falling between 1 and 6 mSv per year, a dose assessment program is necessary, which may involve workplace or individual monitoring. However, when RDE is anticipated to exceed 6 mSv annually, individual monitoring of transportation personnel becomes mandatory.

#### Data integration and mapping

In our study, we utilize three primary criteria to identify potential mineralization zones. Initially, we examine key alteration minerals such as iron oxides, chlorite, kaolinite, sericite, montmorillonite/illite, and alunite, which serve as indicators of mineralization-related alteration processes. Additionally, we assess the density of lineaments across the study area. These lineaments, representing linear surface features, often align with geological structures conducive to mineralization, such as faults and fractures. Furthermore, we consider zones exhibiting elevated F-parameter values exceeding 10. This parameter, reflecting the intensity of alteration processes, aids in pinpointing areas with significant mineralization potential. By integrating these criteria, we aim to accurately map the most promising mineralization zones within our study area.

In terms of environmental mapping, our analysis extends to mapping radiological hazards within the study area. Through exposure and dose rate analyses, we evaluate the environmental impact of natural radiation resulting from the anomalous distribution of natural radioelements and mineral exploration activities. This mapping endeavor is valuable for understanding the extent of radiological risks and assessing potential environmental implications associated with these activities.

## Results

### Spectral enhancement for highlighting lithology

#### False color composite (FCC)

**Fig. 4:**
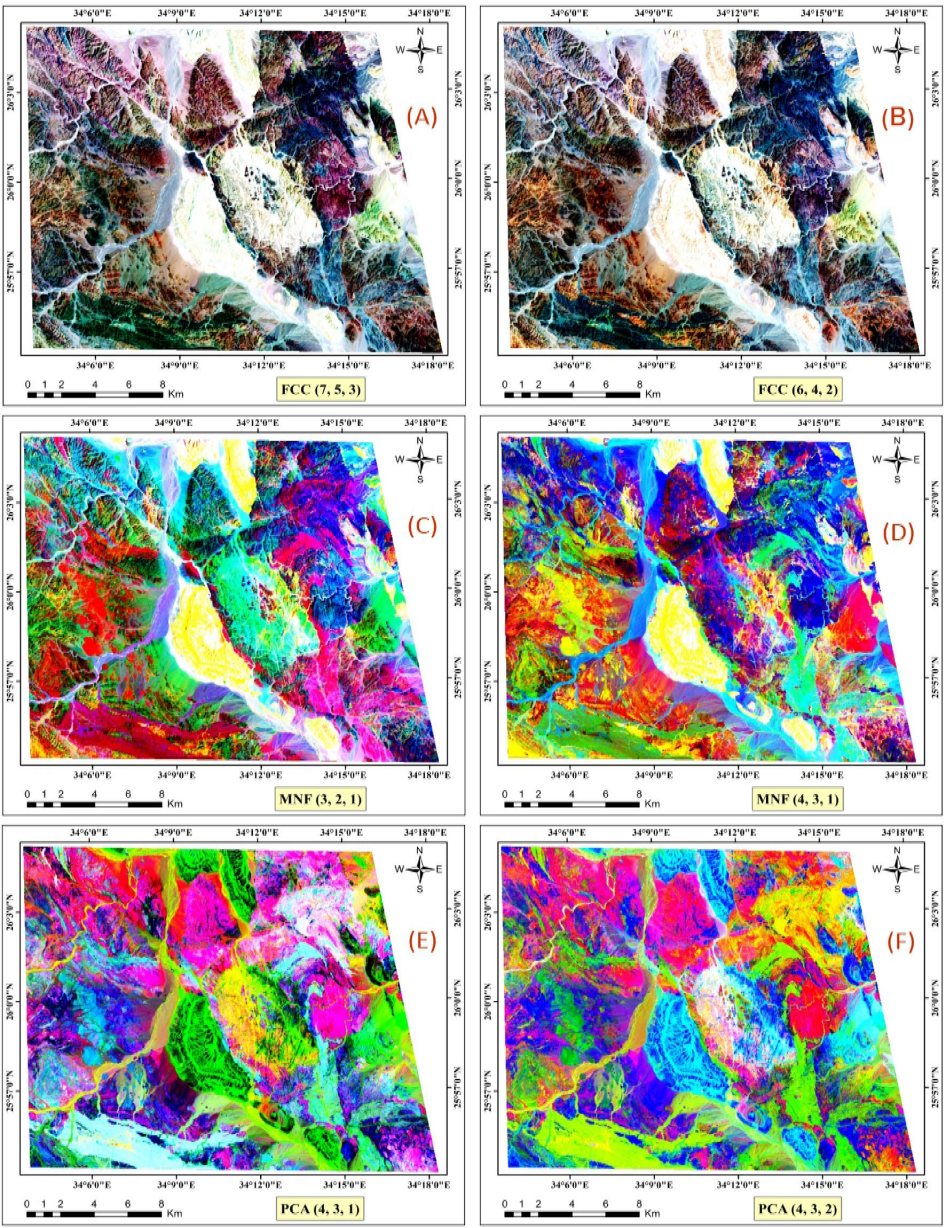
Landsat-9 images displayed in various combinations: FCC images (7-5-3) and (6-4-2) in RGB, MNF images (3-2-1) and (4-3-1) in RGB, and PCA images (PC4-PC3-PC1) and (PC4-PC3-PC2) in RGB, respectively. (By ENVI v.5.3. https://www.l3harrisgeospatial.com/Software-Technology/ENVI).

The color composite method utilizes spectral bands, incorporating red (R), green (G), and blue (B) channels to represent multispectral data. By combining bands in the visible and infrared spectrum, this enhancement is effectively achieved^43^. The FCC technique further enables the rapid and efficient differentiation of main lithological features^53^. This approach enhances the visualization and interpretation of geological and mineralogical characteristics in remote sensing data, as illustrated in Fig. 4A and B. The figure displays an FCC image using three bands (7, 5, and 3) and (6, 4, and 2), respectively, efficiently differentiating the main lithological features.

#### Minimum noise fraction (MNF)

The MNF is an image processing technique that transforms original multispectral image bands into a new set called MNF components^42^. The primary goal of MNF is to maximize the signal-to-noise ratio, effectively enhancing pertinent information while minimizing noise impact^24^. Widely used to improve interpretability and feature discrimination in remote sensing data, MNF finds applications in geological mapping. Figure 4C and D display MNF images using MNF components (3, 2, and 1) and (4, 3, and 1), respectively, efficiently differentiating the main lithological units.

#### Principal component analysis (PCA)

The principal component transformation is a widely employed multivariate statistical technique in image processing for geological mapping, transforming correlated spectral bands into a reduced set of uncorrelated spectral bands known as principal components (PCs)^44,54^. Applied to multispectral remote-sensing images, PCA highlights spectral responses related to specific minerals resulting from hydrothermal alteration processes^55^. In our study, using ENVI software, PCs were calculated for the Landsat-9 VNIR-SWIR seven bands, and composite images were generated using PC4, PC3, and PC1, as well as PC4, PC3, and PC2, representing the red, green, and blue channels, respectively (Figs. 4E,F). Additionally, Table 4 illustrates the PCs derived through PCA on seven bands, presenting coefficients for each band in the corresponding PC. This table provides details on eigenvectors, eigenvalues, and the percentage of total variance explained by each PC. Eigenvalues and eigenvectors are fundamental as eigenvalues quantify the amount of variance captured by each principal component, indicating how much information (variance) each component holds, while eigenvectors represent the directions in the feature space along which the data varies the most, forming the principal components^44,54^. During PCA, eigenvectors are computed from the covariance matrix of the data. The eigenvector with the highest eigenvalue points in the direction of maximum variance and forms the first principal component, with subsequent eigenvectors capturing the remaining variance in descending order. Thus, eigenvalues and eigenvectors are essential in lithology for reducing data complexity, identifying key geological features, and enhancing the interpretation and visualization of lithological variations^53,55^. PC1, with the highest eigenvalue and percentage, captures the most significant variance in the data, followed by subsequent components in descending order of importance. The PCA plays a valuable role in comprehending each band's contribution to the overall dataset variance, facilitating the identification of the most influential components. Consequently, the application of the PCA processing method proves to be efficient in distinguishing the lithological units of the study area.

**Table 4:** Eigenvector matrix and eigenvalues of principal component analysis on Landsat-9.

	PC 1	PC 2	PC 3	PC 4	PC 5	PC 6	PC 7
Band 1	− 0.926558	0.356932	0.068675	0.096613	0.000794	0.006262	− 0.000053
Band 2	− 0.365668	− 0.82262	− 0.37176	− 0.20618	0.0867	0.0296	− 0.021908
Band 3	− 0.08249	− 0.32112	0.899325	− 0.24597	− 0.13499	0.047347	0.018161
Band 4	0.016706	0.274335	− 0.05033	− 0.82171	0.495921	0.001509	− 0.027961
Band 5	0.008534	− 0.13195	0.210514	0.432817	0.809823	− 0.30554	− 0.04126
Band 6	0.022025	− 0.0061	0.028447	0.152054	0.268789	0.916867	0.250313
Band 7	− 0.011646	− 0.00871	− 0.02516	− 0.04472	− 0.01619	− 0.25069	0.966462
Eigenvector	0.031148	0.000945	0.000436	0.00012	0.000034	0.000007	0.000001
Eigenvalue %	95.28005	2.890704	1.3337	0.367074	0.104004	0.021413	0.003058946

### Alteration minerals mapping

Alteration minerals serve as key indicators of hydrothermal alteration zones, offering valuable guidance in the search for potential mineralization. The spectral signatures of key alteration minerals, including iron oxides (hematite/goethite), chlorite, kaolinite, sericite, montmorillonite/illite, and alunite, were utilized from the USGS spectral library as reference endmembers. These endmembers were employed to map the distribution of alteration minerals across the study area.

**Fig. 5:**
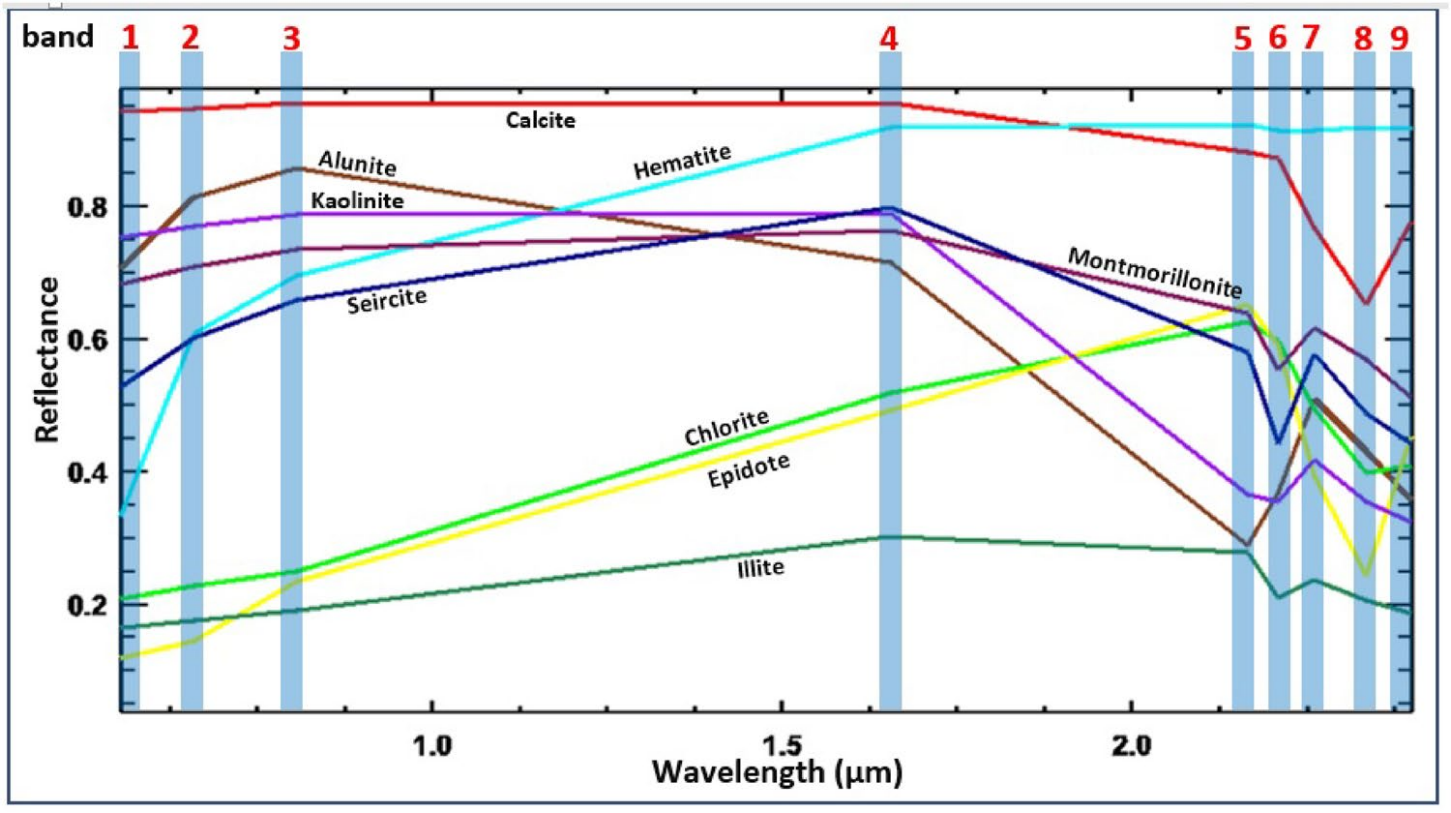
ASTER VNIR-SWIR spectral signatures of endmember minerals spectra, resampled from the USGS spectral library (https://www.usgs.gov/labs/spectroscopy-lab/science/spectral-library). (By ENVI v.5.3. https://www.l3harrisgeospatial.com/Software-Technology/ENVI).

The analysis of USGS standard mineral spectral curves (Fig. 5) concerning ASTER bands provides valuable insights into the spectral characteristics of different alteration zones. Notably, Al–OH minerals, including kaolinite, muscovite, and montmorillonite, which are prevalent in argillic alteration zones, exhibit prominent reflectance in band 4 of the SWIR region^56^. Conversely, the phyllic zone, characterized by sericite (muscovite), displays a distinctive Al–OH absorption feature primarily centered at 2.20 μm (ASTER band 6), accompanied by a secondary feature near 2.38 μm (ASTER band 8). Reflectance spectra within the propylitic zone reveal significant Fe, Mg-OH absorption features, along with CO3 features attributed to molecular vibrations in chlorite, epidote, and carbonate minerals, distinctly observed in the 2.35 μm (ASTER band 8) region^57^.

**Fig. 6:**
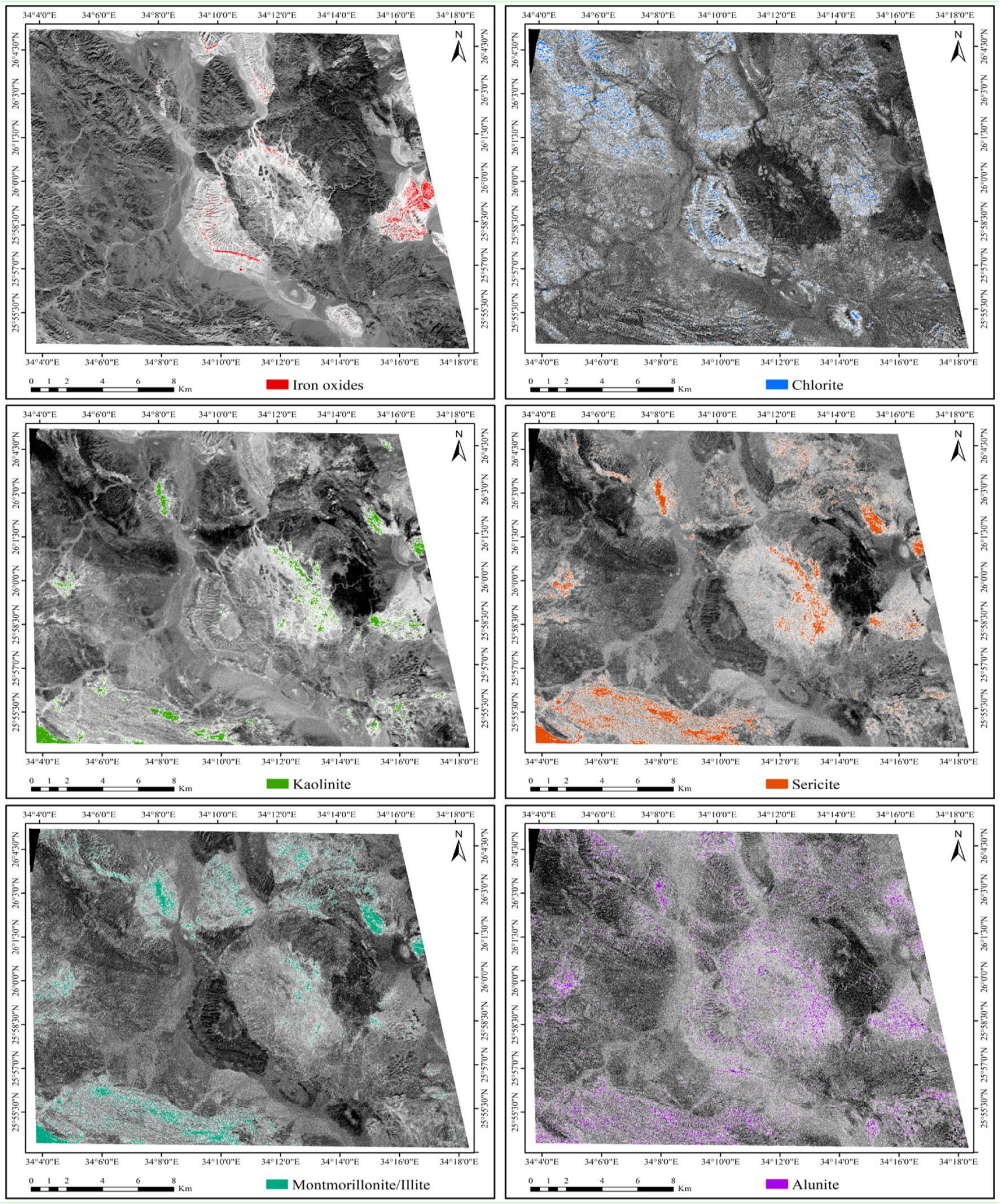
The abundance of alteration minerals across ASTER band ratio images, employing band ratios: gossanised zone (iron oxides: B2/B1), propylitic zone (chlorite: (B7 + B9)/B8), phyllic zone (sericite: (B7/B6)), argillic zone (kaolinite: (B4/B5)*(B8/B6), montmorillonite/illite: (B5 + B7)/B6), and advanced argillic zone (alunite: (B7/B5)*(B7/B8)). (By ENVI v.5.3. https://www.l3harrisgeospatial.com/Software-Technology/ENVI).

Furthermore, to enhance the detection of phyllic alterations characterized by minerals like sericite and muscovite, as well as argillic or advanced argillic alterations containing minerals such as kaolinite, montmorillonite, and illite, and propylitic alterations involving chlorite, epidote, and carbonate minerals, alongside iron oxides like goethite, hematite, and limonite, various band ratios were utilized, as presented in Fig. 6. The ASTER band ratio (B2/B1) serves as an indicator for iron oxides, as noted in several studies^46–48^. Iron oxide minerals are predominantly characterized by their spectral signatures in bands 1 and 3^44^. These minerals exhibit distinct absorption features in bands 1 and 2, coupled with reflectance features in band 4^16,58^. The ASTER band ratio (B7 + B9)/B8 is a reliable indicator of chlorite, as minerals containing magnesium hydroxide and carbonates, such as chlorite, epidote, and carbonate, exhibit distinct spectral responses in band 8. This enhances the capabilities of ASTER data in mineral detection and characterization^44^. Additionally, specific ASTER band ratios are utilized for different alteration minerals. For kaolinite, the ratio (B4/B5)*(B8/B6) is employed^49^, while for montmorillonite/illite, the ratio (B5 + B7)/B6 is utilized^48^. Moreover, for alunite, the ratio (B7/B5)*(B7/B8) is applied^49^. These ratios help identify minerals like kaolinite, illite, montmorillonite, and alunite, which display an Al–OH absorption feature near 2.20 μm. It's worth noting that kaolinite and alunite minerals exhibit significantly different spectral curves compared to muscovite/illite minerals^59^. Specifically, kaolinite shows a secondary feature at 2.17 μm, while alunite exhibits a minimum at 2.17 μm corresponding to ASTER band 5 instead of 2.20 μm^16,44,59,60^. Furthermore, the ASTER band ratio (B7/B6) emerges as a dependable indicator of hydrothermal alteration, particularly in enhancing the detection of sericite, a mineral rich in hydroxyl groups. This reliability stems from the distinct spectral signatures exhibited by sericite, characterized by heightened reflectance in band 4 and diminished reflectance in band 6^44^. Moreover, these minerals manifest an absorption feature in band 6, resulting in a notable contrast between the reflectance levels in bands 4 and 7 compared to band 6^44,61^.

### Structural features mapping

**Fig. 7:**
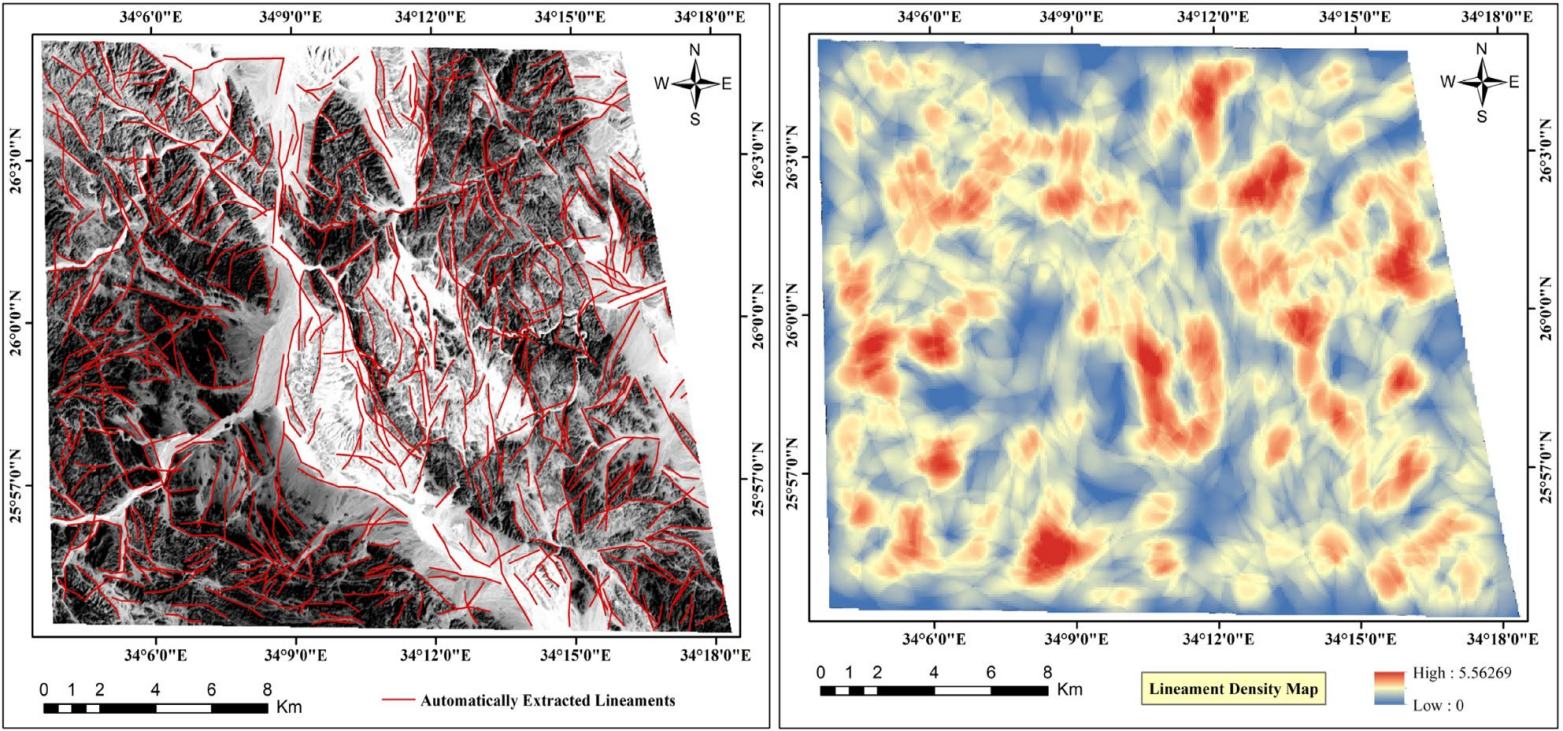
Automatically extracted lineaments and density map of Jabal Hamadat area. (By PCI Geomatica https://catalyst.earth/about/).

The presence and characterization of lineaments significantly influence mineral exploration endeavors, serving as vital indicators of subsurface geological structures and potential mineral deposits^62^. These linear features comprising faults, fractures, and joints, serve as pathways for mineral-rich fluids, enabling their migration and subsequent deposition^63^. By mapping and analyzing lineaments, we can identify zones of structural weakness and fluid flow pathways that are conducive to mineralization. This approach enables us to pinpoint favorable locations for ore deposits^50^. The lineaments were automatically mapped using the "LINE" module within the "PCI Geomatica" software, employing a shaded relief image derived from PCA data covering the study area. A lineament density map was generated (Fig. 7), where regions exhibiting high lineament density are considered promising sites for abundant faults and fractures. These structural features serve as conduits for subsurface fluids, including hydrothermal solutions, facilitating their migration and thereby influencing the formation of hydrothermally altered zones^24,50^.

### Airborne radiometric data analysis

#### Description of the radioelements distribution

**Fig. 8:**
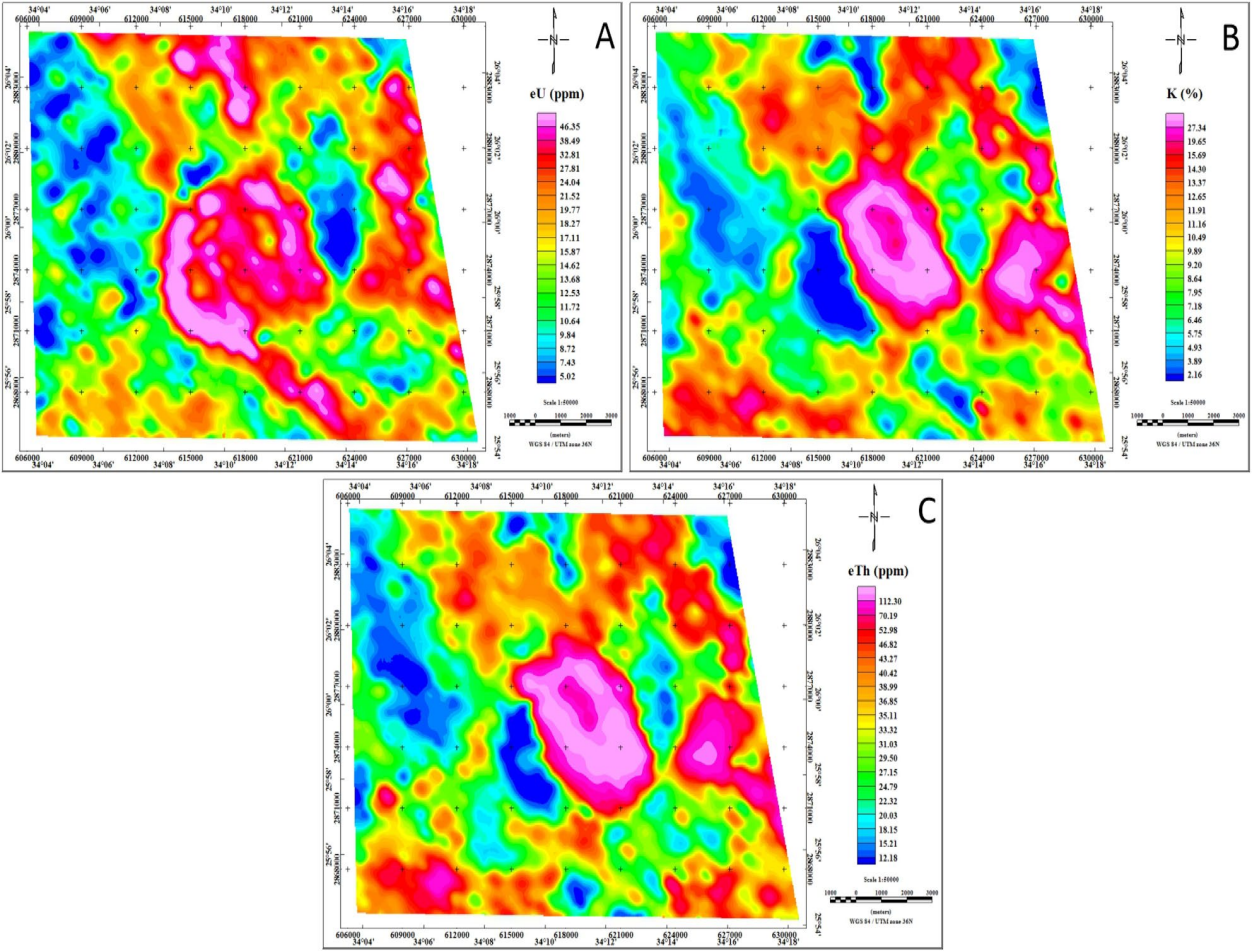
Spatial distribution of eU (ppm), K (%), and eTh (ppm) concentrations of the Jabal Hamadat area, (map values*10). (By Geosoft Oasis Montaj software https://www.seequent.com/products-solutions/geosoft-oasis-montaj/).

The airborne radiometric survey conducted in the study area yielded valuable insights into three key variables: an equivalent uranium contour map (eU, in ppm), an equivalent thorium contour map (eTh, in ppm), and a potassium contour map (K40, in %), as depicted in Fig. 8. These maps elucidate the spatial distribution of concentrations of these radiometric elements, offering a detailed portrayal of the lateral variability of different rock and soil types in terms of surface elemental concentration^41^. The presented figure provides a comprehensive visualization of the elemental composition across the study area. The distribution of radiometric elements in the study area delineates three distinct concentration levels: the first level, spanning from bright magenta to heavy magenta, denotes the highest concentrations; the second level, ranging from bright green to yellow, represents intermediate values; and the third level, varying from blue to heavy green, signifies the lowest concentrations.

An analysis of the equivalent uranium radiation map (Fig. 8A) reveals three distinct concentration levels based on uranium content. The first level, characterized by higher concentrations ranging from 2.5 to over 9.2 ppm, is primarily associated with Younger granitoids, Thebes, Esna, Dakhla, Duwwi, Nubia formations, and certain parts of the Ophiolite group in the central part of the study area. Moderate uranium concentrations, ranging from 1 to 2.5 ppm, are identified in Dukhan volcanic, Hammamat sediments, Calc-Alkaline volcanic group, and Schist group. Conversely, lower uranium concentrations, below 1 ppm, are observed in some sections of the Ophiolite group and Schist group on the western side of the study area.

An examination of the potassium radiation map (Fig. 8B) reveals distinct patterns in potassium concentrations. The highest potassium values, representing the first level, are predominantly associated with Younger granitoids, Dukhan volcanic, and Hammamat sediments, ranging between 1.5% and exceeding 3.5%. Intermediate values, constituting the second level, are observed in the Schist group and Calc-Alkaline volcanic group, ranging from 0.6% to 1.5%. Conversely, the third zone, characterized by lower potassium concentrations below 0.6%, is closely linked to Thebes, Esna, Dakhla, Duwwi, and Nubia formations. Additionally, the Ophiolite group exhibits intermediate to low concentrations.

The equivalent thorium radiation map (Fig. 8C) reveals three distinct levels of thorium concentrations across the study area. The highest level, ranging from 4.5 to over 20 ppm, is primarily found in Younger granitoids, Hammamat sediments, and certain areas of Dukhan volcanic in the northeastern part of the study area. Intermediate values, ranging from 2.1 to 4.5 ppm, characterize the second level and are predominantly associated with the Calc-Alkaline volcanic group, Schist group, and Ophiolite group. The lowest level of thorium concentration, below 2.1 ppm, is observed in Thebes, Esna, Dakhla, Duwwi, and Nubia formations, as well as some sections of the Ophiolite group in the western part of the study area.

#### Analysis of the radioelement ratios

**Fig. 9:**
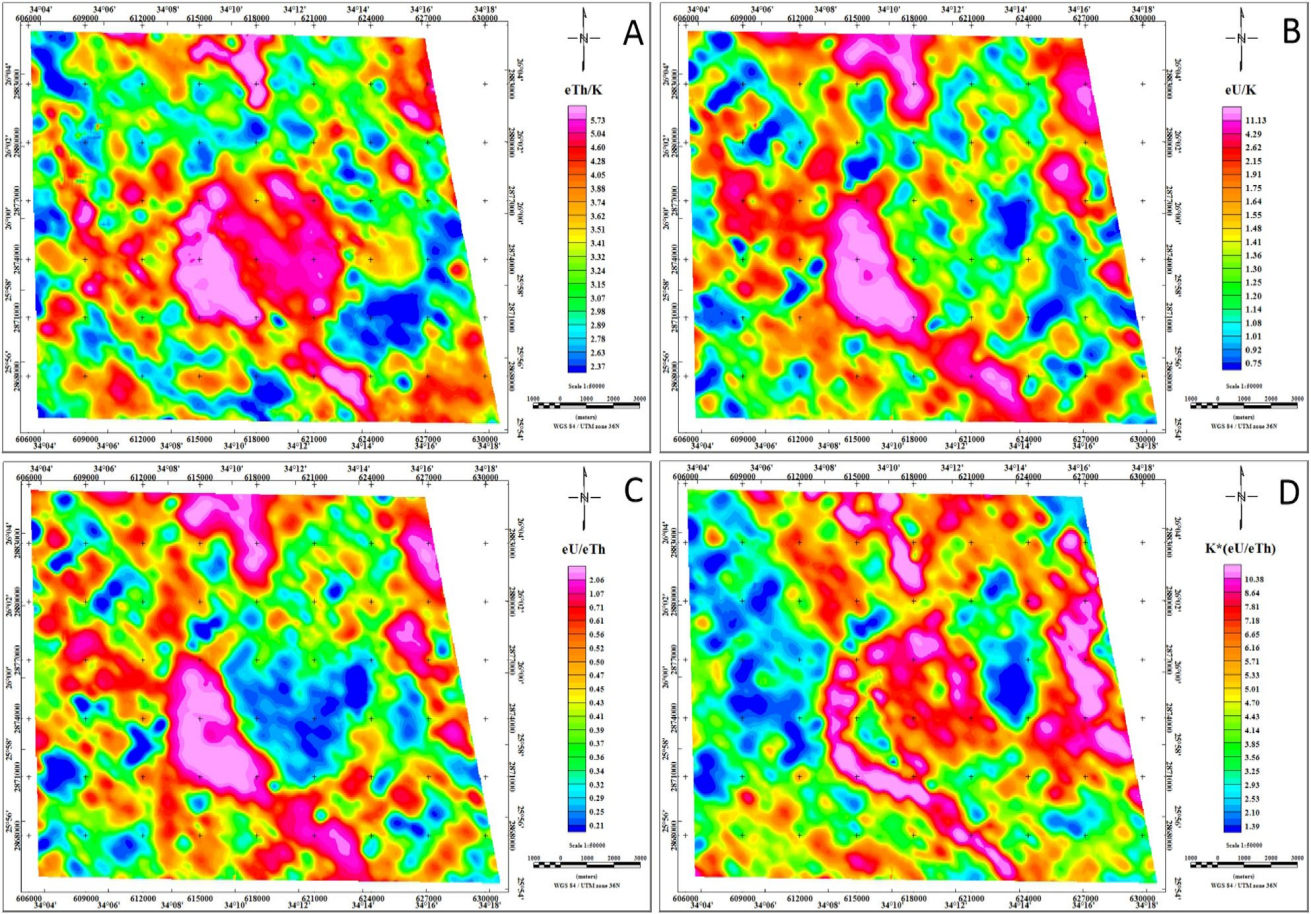
Radioelement ratios maps eTh/K, eU/K, eU/eTh, and K*(eU/eTh) of the Jabal Hamadat area. (By Geosoft Oasis Montaj software https://www.seequent.com/products-solutions/geosoft-oasis-montaj/).

The radioelement ratios play an indispensable role in mineral exploration, offering valuable insights into the concentration relationships among specific radioelements. Key ratios such as eTh/K, eU/K, and eU/eTh are widely employed to delineate mineralization zones effectively (Fig. 9). Analyzing these ratios enables the identification of potential mineral deposits and the tailoring of exploration strategies accordingly.

The radioelement ratio map (eTh/K) serves as a powerful tool for mineralization detection, providing a nuanced perspective by assessing the ratio of eTh to K concentrations (Fig. 9A). Elevated eTh/K ratios serve as indicators of potential mineralization zones, facilitating a targeted approach to identifying areas of geological significance^9,64^. The eTh/K ratio exhibits notable variations across different geological formations in the study area. Particularly, younger granitoids, Thebes, Esna, Dakhla, Duwwi, and Nubia formations, as well as Hornblende-quartz diorite and certain sections of the Ophiolite group, display remarkably high eTh/K ratio values, ranging from 4.28 to over 5.73. This signifies a substantial concentration of thorium relative to potassium in these formations. Conversely, Hammamat sediments, Dukhan volcanic rocks, and specific areas within the Ophiolite group show intermediate eTh/K ratio values, ranging from 2.95 to 4.28, indicating a moderate concentration level. In contrast, some segments of the Calc-Alkaline volcanic group and Schist group exhibit eTh/K ratio values below 2.95, suggesting a relatively lower thorium concentration.

The eU/K ratio map serves as a valuable tool for mineral exploration, effectively pinpointing areas with heightened uranium concentrations in relation to potassium (Fig. 9B). Notably, formations such as Thebes, Esna, Dakhla, Duwwi, and Nubia, as well as Hornblende-quartz diorite, exhibit elevated eU/K ratio values ranging from 2.15 to over 11.13, indicating a notable enrichment in uranium content. The Dukhan volcanic rocks, Hammamat sediments, and Calc-Alkaline volcanic group display values ranging between 1.14 and 2.15, signifying a moderate concentration of uranium relative to potassium. Certain parts of the Ophiolite group and Schist group register eU/K ratio values below 1.14, suggesting a comparatively lower uranium concentration in these specific areas.

The eU/eTh ratio map visually represents the relationship between eU and eTh concentrations, offering valuable insights into uranium enrichment (Fig. 9C). Remarkably, the uranium enrichment observed on the eU/eTh ratio map aligns with the periphery of the potassium anomaly^65^. The most significant eU/eTh ratio anomaly, ranging from 0.65 to values exceeding 2.06, is intricately linked to the strongly altered mineralized zone within the Thebes, Esna, Dakhla, Duwwi, and Nubia formations, as well as Hornblende-quartz diorite, and specific sections of the Ophiolite group and Dukhan volcanic in the study area.

#### The F-parameter approach

The identification of mineralized alteration zones, involves a normalizing approach, calculated using the equation (K*(eU/eTh))^66^. This methodology relies heavily on the F parameter, a pivotal metric proven effective in delineating potassic alterations associated with mineralization, with values exceeding 10 typically indicative of altered rocks^24,67^. In the Jabal Hamadat area (Fig. 9D), specific zones within the Schist group and Ophiolite group exhibit F-parameter values below 3, signaling the lowest level of alteration. Meanwhile, the Calc-Alkaline volcanic group, Hammamat sediments, Dakhla formation, and certain areas of the Schist group fall within the 3 to 10 range which indicating an intermediate degree of alteration. Particularly noteworthy are the Younger granitoids, selected areas within the Dukhan volcanic and Ophiolite group, and formations like Thebes, Esna, Duwwi, and Nubia, showcasing F-parameter values exceeding 10, suggesting intense alteration processes. By delineating regions with elevated F-parameter anomaly values, this method effectively highlights zones likely to potential mineralization. Consequently, the F-parameter emerges as a valuable tool for identifying potential mineralization areas strongly influenced by potassium alterations in the study area.

### Potential mineralization zones mapping

**Fig. 10:**
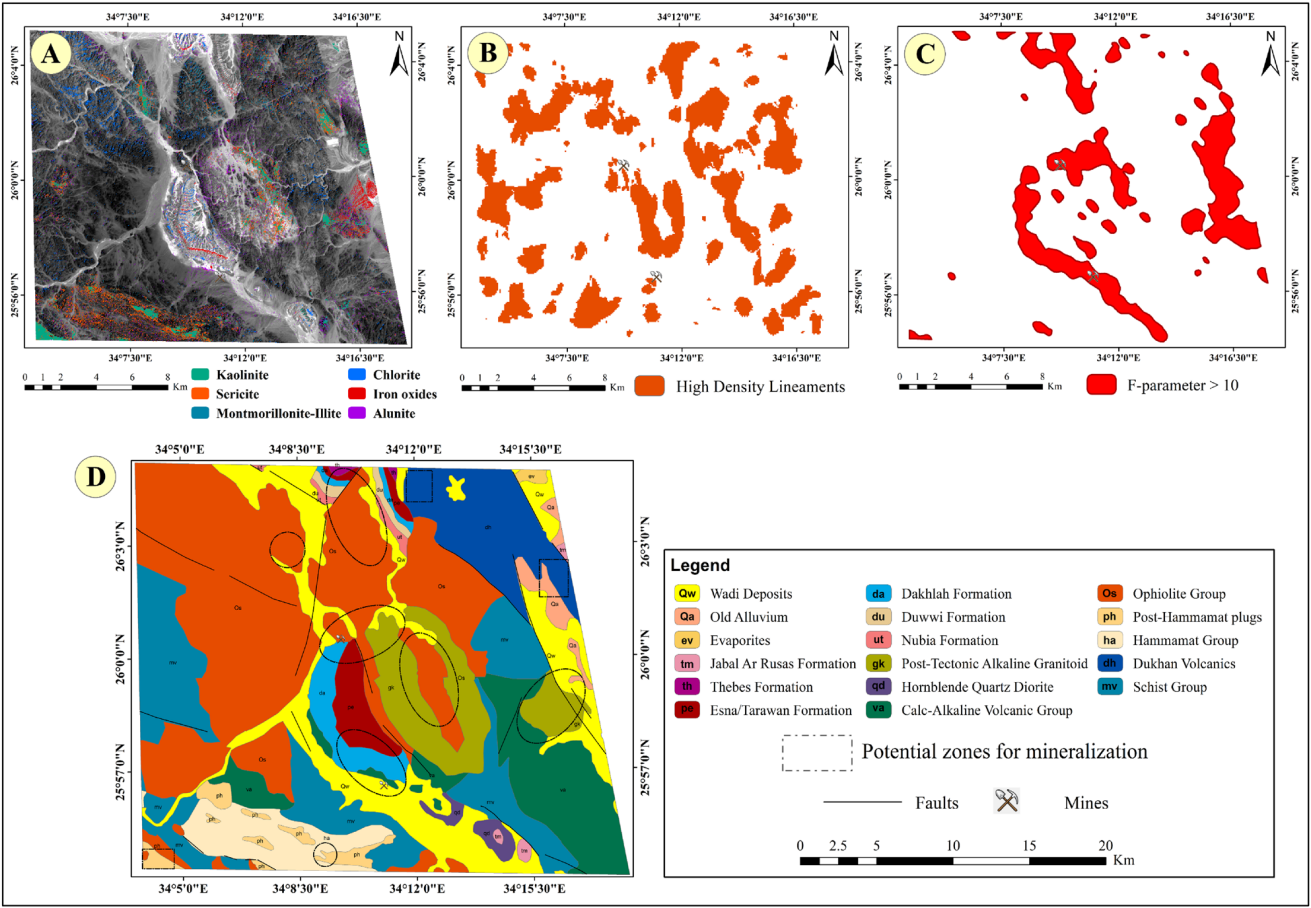
(**A**) The abundance map for all key alteration minerals (kaolinite, sericite, montmorillonite-illite, chlorite, iron oxides, alunite) resulted from the BR technique, (**B**) High-density lineaments map, (**C**) The "F-parameter > 10 zones" map, (**D**) The potential zones for mineralization overlaid on the geological map of the Jabal Hamadat area. (By ArcGIS v.10.5. https://www.esri.com/en-us/arcgis/products/arcgis-desktop/overview/).

Following the mapping of hydrothermal alteration minerals and/or mineralization zones, key indicators such as iron oxides (hematite/goethite), chlorite, kaolinite, sericite, montmorillonite/illite, and alunite were extracted, leading to the identification of a definitive number of alteration zones, as illustrated in Fig. 10. Subsequently, high lineament density areas were determined, indicating promising zones characterized by abundant faults and fractures. These structural features act as conduits for subsurface fluids, including hydrothermal solutions, facilitating their migration and influencing the formation of hydrothermally altered zones. Next, zones exhibiting F-parameter values exceeding 10, indicating intense alteration processes and identifying potential mineralization areas strongly influenced by potassium alterations were defined.

**Fig. 11:**
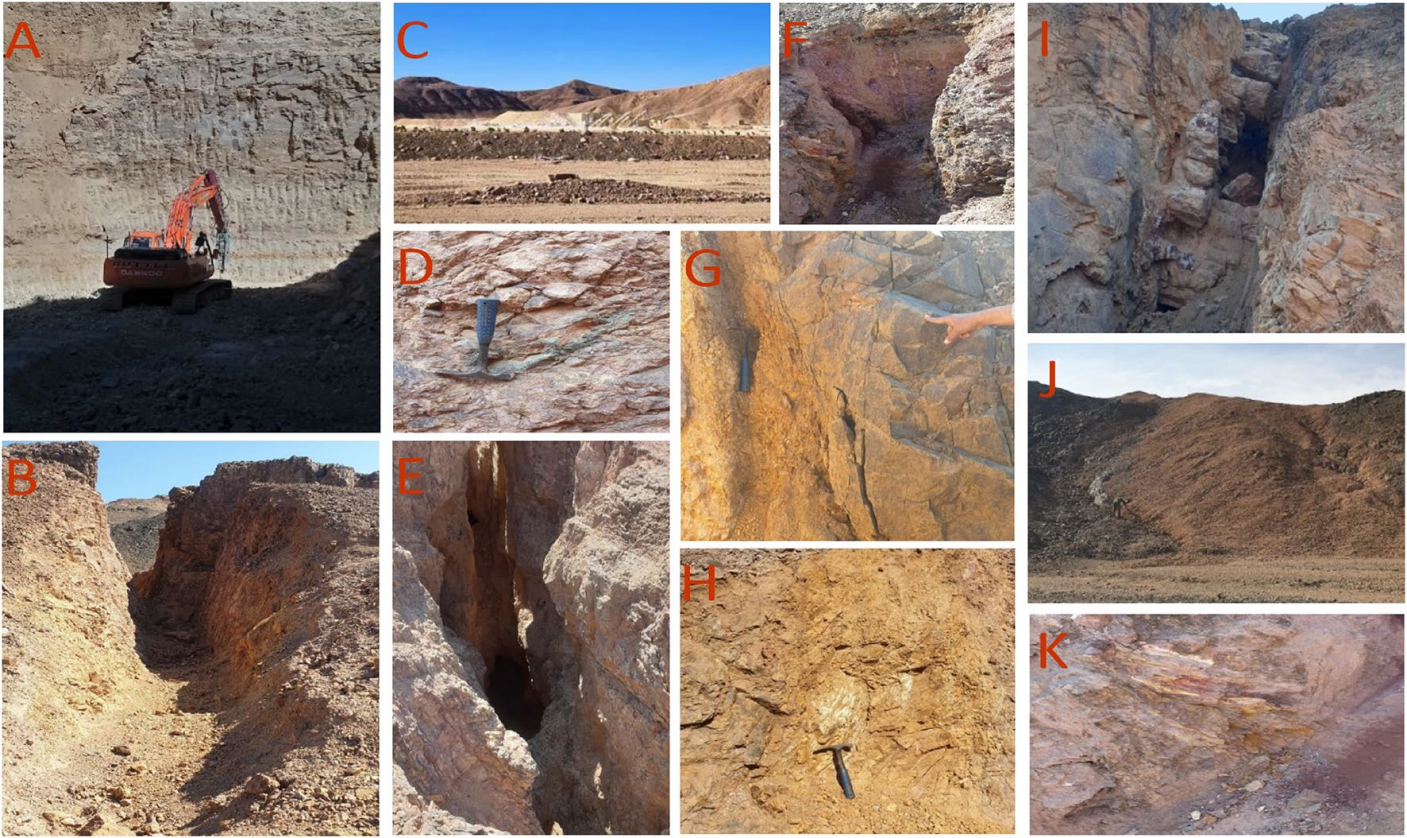
Signs of mining work and highly potential localities for mineralization observed during the field investigation; (**A**) the succession of mine, starting from top by gypsum layer from 2 m up to 25 m thickness in some places underlained by black shale up to 8 m then the phosphate beds with cumulative thickness about 2.5 m, (**B**) tunnel in the alteration zones, (**C**) marly limestone and phosphate work, (**D**) alternations of phosphate beds in the limestone host rocks, (**E**) excavated work in the alteration zones, (**F**) ultramafic rocks including some listwaenite ridges enriched in hematite and limonite, (**G**) alterations in the metavolcanic rocks, and (**H**) associations of phosphate beds and patches of oil shale bed, (**I**) quartz veins enriched in iron oxides, (**J**) felsite plug in the metavolcanis, (**K**) ultramafic rocks enriched in iron oxides.

Finally, a comprehensive approach was employed by combining zones characterized by common features such as alteration minerals abundance, high density lineaments, and elevated F-parameter values exceeding 10. By integrating these criteria, the most promising zones for mineralization were delineated, optimizing the selection process for further exploration and resource allocation, as presented in Fig. 11.

### Radiological hazard assessment

Radiological hazard assessments utilize measurements of radiation exposure rate and radiation dose rate to comprehensively evaluate the potential risks associated with radioactive sources in a given area. Radiation exposure rate provides real-time data on the amount of radiation present in the environment at specific locations. This measurement aids in identifying areas with elevated radiation levels, highlighting potential sources of contamination, and pinpointing areas requiring remediation efforts^29,33,64^. Conversely, radiation dose rate offers insights into the cumulative radiation absorbed by organisms over time. By analyzing radiation dose rates, assessments can estimate the long-term health risks posed by exposure to radioactive materials, informing decision-making processes related to environmental management and public health protection^32,34,68^. Together, these measurements form the foundation of effective environmental impact assessments, guiding the development of mitigation strategies and regulatory measures to safeguard both ecosystems and human populations^52^.

**Fig. 12:**
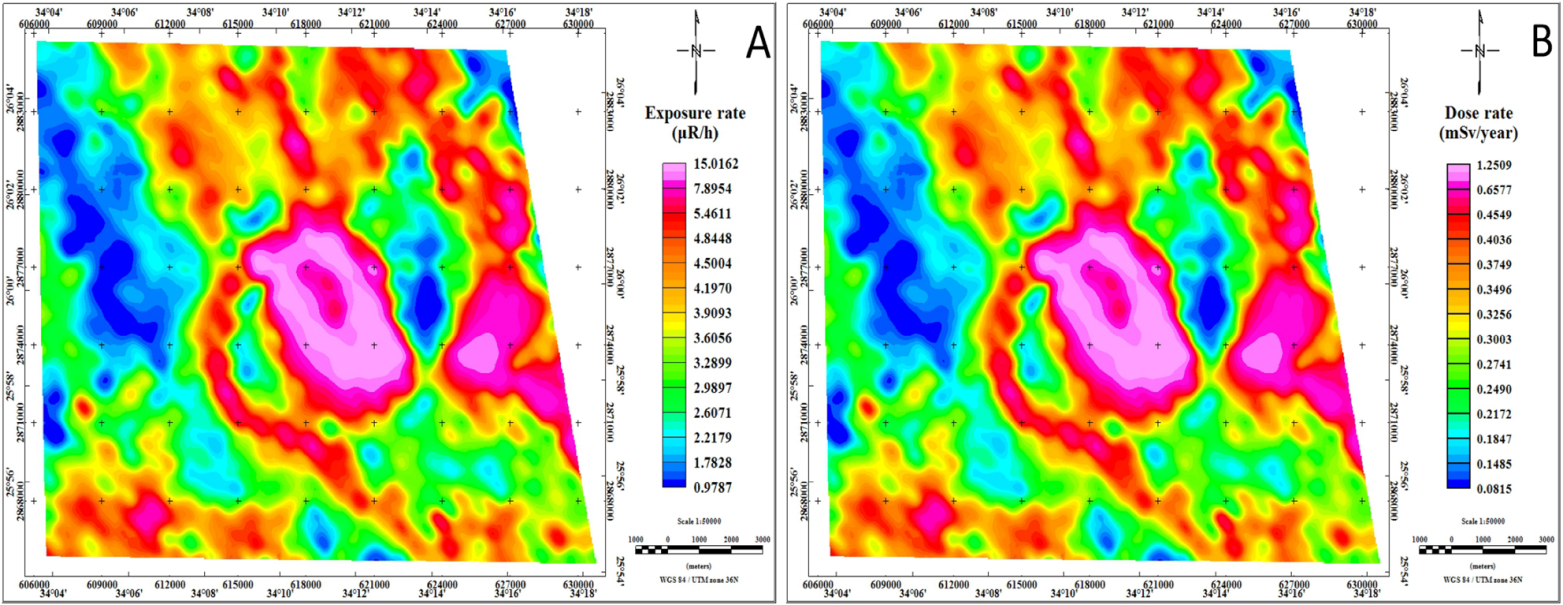
Exposure rate and radiation dose rate maps of the Jabal Hamadat area. (By Geosoft Oasis Montaj software https://www.seequent.com/products-solutions/geosoft-oasis-montaj/).

The radiation exposure rate map (Fig. 12A) reflects three distinct levels of radiation intensity. The lowest level, characterized by readings below 2.4 μR/h, is depicted in blue and is associated with the Schist group and Ophiolite group. The intermediate level, ranging from 2.4 to 4.8 μR/h, is represented by varying colors from green to yellow and orange, covering the Calc-Alkaline volcanic group, Dukhan volcanic, Hammamat sediments, as well as Thebes, Esna, Dakhla, Duwwi, and Nubia formations. The highest radiation level, exceeding 4.8 μR/h, is depicted in red to magenta on the RER map, indicating localized anomalies primarily linked to Younger granitoids and certain areas within the Dukhan volcanic and Ophiolite group.

The dose rate map (Fig. 12B) provides a visual representation of the radiation levels resulting from terrestrial gamma radiation within the study area. This map divides the radiation levels into three distinct levels based on their intensity. The lowest level, characterized by levels below 0.2 mSv/year, predominantly corresponds to areas associated with the Ophiolite group geological formation. The intermediate level, spanning from 0.2 to 1.0 mSv/year, encompasses a broader range of geological formations, including Thebes, Esna, Dakhla, Duwwi, and Nubia formations, as well as the Calc-Alkaline volcanic group, Dukhan volcanic, Hammamat sediments, Hornblende quartz diorite, and the Schist group. Conversely, the highest level, with radiation levels exceeding 1.0 mSv/year, is exclusively observed over the Younger granitoids located in the central and eastern regions of the study area.

## Discussion

The integration of multispectral remote sensing data, including Landsat 9 and ASTER, with airborne geophysical data proves powerful to augment the precision of lithological unit characterization and the mapping of potential mineralization zones^14,21,25,36^. This is due to the complementary nature of sensor specifications, covering a wide and specific portion of the spectrum, which enables the characterization of lithological rock units, lineaments, and alteration minerals. Various minerals exhibit characteristic absorption and reflection features in specific spectral bands. Multispectral sensors are capable of detecting these spectral features, allowing for the identification of specific minerals associated with alteration zones^11,23,50,69^. Additionally, airborne radiometric data can detect subtle variations in the radioactivity of rocks caused by the presence of alteration minerals associated with hydrothermal alteration zones. These alteration minerals often contain radioactive elements such as uranium, thorium, and potassium, which emit gamma rays that can be detected by radiometric sensors^24,65,67^.

Recent advancements in the application of image processing techniques on multispectral remote sensing data have proven highly successful in mapping and distinguishing lithological units as well as identifying hydrothermal alteration patterns^5,12,22,70,71^. In the present study, we employed spatial and spectral analysis techniques to analyze Landsat-9 multispectral data, employing methods such as FCC, MNF, and PCA to enhance our understanding of lithological characteristics. Our investigation extended to ASTER satellite data analyses, where we employed BR technique to identify the key alteration minerals, including kaolinite, sericite, montmorillonite-illite, chlorite, iron oxides, and alunite, across the study area. Additionally, automatic extraction of lineaments was conducted, providing insights into zones of deformation and potential channels for hydrothermal solutions that may carry valuable mineral resources.

The airborne radiometric data emerges as a potent tool for the identification, mapping, and characterization of alteration zones, playing a pivotal role in mineral exploration efforts^23,27,28,36,72–74^. In the context of the present study, this dataset provides essential insights into the spatial distributions and concentrations of radioelements in the Jabal Hamadat area, including K in percentage, eU in ppm, and eTh in ppm. The study leverages various radioelement ratios, such as eTh/K, eU/K, eU/eTh, and (K*(eU/eTh)), to effectively delineate hydrothermal alteration zones. The comprehensive analysis of airborne radiometric data enhances our understanding of the geological characteristics, aiding in the targeted exploration of areas with significant mineral deposit potential.

By utilizing a comprehensive approach, we integrated three key components. Firstly, we combined data on hydrothermal alteration minerals, including iron oxides, chlorite, kaolinite, sericite, montmorillonite/illite, and alunite. Secondly, we identified areas with dense lineaments, indicating zones rich in faults and fractures, which act as pathways for subsurface fluids like hydrothermal solutions, thus influencing altered zone formation. Thirdly, we located zones with F-parameter values exceeding 10, signifying intense alteration processes and potential mineralization zones. This innovative strategic fusion resulted in the identification of ten promising zones for mineral exploration.

The exposure and dose rates were determined to assess the environmental impact of natural radiation and evaluate potential hazards resulting from the anomalous distribution of natural radioelements and mineral exploration works within the study area. This method serves as a powerful tool in environmental mapping, offering valuable insights into the spatial distribution and intensity of radiation hazards in the environment^29,32–34^. Through this approach, a comprehensive understanding of radiation risks is gained, enabling the development of effective mitigation strategies to safeguard human health and the ecosystem^52,64,68,75,76^. Consequently, our investigation revealed that the study area has surpassed the maximum allowable safe radiation rate, with radiation levels exceeding 1.0 mSv/year in specific areas, notably evident over the younger granites located in the central and eastern parts. This finding raises concerns regarding potential health risks associated with elevated radiation exposure in these regions. According to the IAEA (2005) classifications of radiation dose equivalents, necessary action is typically required within this dose range to evaluate and control worker doses, as it is recommended that no individual should receive more than 1.0 mSv/year^52^. However, it remains imperative to maintain vigilance and closely monitor radiation levels to ensure ongoing safety and compliance with regulatory standards.

**Fig. 13:**
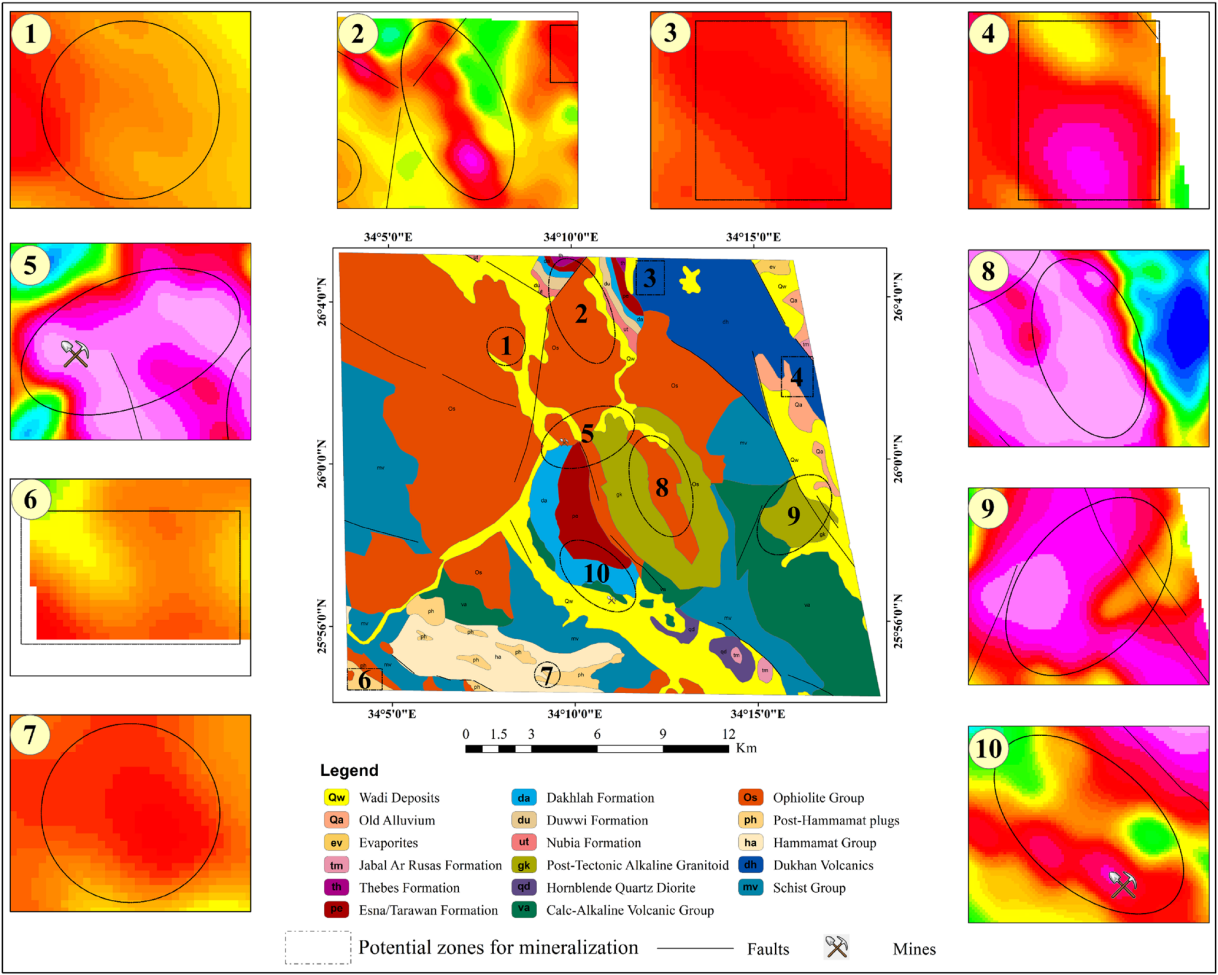
The top ten potential mineralization sites and their corresponding radiological hazards at the Jabal Hamadat area. (By ArcGIS v.10.5. https://www.esri.com/en-us/arcgis/products/arcgis-desktop/overview/).

In our investigation, we identified specific areas with elevated radiological hazards, focusing particularly on potential mineralization zones and the Jabal Hamadat mining sites, as illustrated in Fig. 13. For example, area 1 exhibited an average dose ranging from 0.35 to 0.48 mSv/year, while area 2 registered levels ranging from 0.26 to 0.75 mSv/year. Similarly, areas 3, 4, 6, and 7 showed radiation levels ranging from 0.39 to 0.48 mSv/year, 0.34 to 0.71 mSv/year, 0.34 to 0.47 mSv/year, and 0.39 to 0.49 mSv/year, respectively. Notably, areas 5, 8, and 9 demonstrated relatively higher radiation levels, with doses ranging from 0.5 to 1.25 mSv/year. Furthermore, our assessment of the mining sites revealed elevated radiation values, with peak readings recorded in area 5 at 1.25 mSv/year and area 10 at 0.64 mSv/year. These findings are significant given that mining activities can lead to the release of naturally occurring radioactive materials from the earth's crust, including radioactive isotopes of uranium, thorium, and their decay products. Consequently, there is heightened concern regarding the exposure of miners and workers to elevated radiation levels during mining operations, which can occur through inhalation of radioactive dust particles, ingestion of contaminated water or food, and direct contact with radioactive materials. To address these risks, several actions can be taken to ensure worker safety and minimize radiation exposure. Overall, these findings emphasize the critical need for comprehensive monitoring and mitigation strategies to address the radiological risks associated with mineralization zones and mining activities in the Jabal Hamadat area.

## Conclusion

This study presents new insights into the integration of multispectral remote sensing data, including Landsat 9 and ASTER, with airborne geophysical data, for mapping potential mineralization zones and assessing the environmental impacts within the Jabal Hamadat area. Employing techniques such as FCC, MNF, and PCA analyses, we gained valuable insights into lithological units. Furthermore, utilizing the BR technique facilitated the identification of alteration mineral indicators, such as iron oxides, chlorite, kaolinite, sericite, montmorillonite/illite, and alunite, which serve as key markers of mineralization-related alteration processes. The analysis of airborne radiometric data, incorporating radioelement ratios (eU/eTh, eU/K, eTh/K) and the F-parameter (K*(eU/eTh)), provided essential information for accurately delineating hydrothermal alteration zones. By adopting a comprehensive approach that considers alteration mineral abundance, high-density lineaments, and elevated F-parameter values exceeding 10, we successfully outlined the most promising mineralization zones. Moreover, our study identified specific areas within the study area surpassing the maximum safe radiation threshold (1.0 mSv/yr), raising concerns regarding radiation exposure for workers, particularly in mining activities. We recommend relocating worker residences away from younger granites and potential mineralization sites, especially the Jabal Hamadat mining sites, which exhibit relatively high dose rates exceeding the recommended levels (average dose of 1.25 mSv/yr). Additionally, the establishment of rigorous monitoring and mitigation measures is imperative to safeguard worker well-being and minimize radiation exposure. These findings provide valuable reference data for monitoring potential radioactivity pollution in the future.

## Data Availability

Data sets generated during the current study are available from the corresponding author on reasonable request.
